# The Applied Genomics Development Strategy by the Croatian Academy of Sciences and Arts paves the way for the future development of applied genomics in Croatia

**DOI:** 10.3325/cmj.2024.65.297

**Published:** 2024-06

**Authors:** Filip Sedlic, Jadranka Sertić, Alemka Markotić, Dragan Primorac, Anita Slavica, Lada Zibar, Kristian Vlahoviček, Vesna Kušec, Ivo Barić, Vladimir Paar, Fran Borovečki, Ljiljana Žmak, Ivan-Christian Kurolt, Nina Canki-Klain, Sunčana Roksandić, Iva Rinčić, Hrvoje Jurić, Vedrana Škaro, Damir Marjanović, Petar Projić, Damir Primorac, Antonio Starčević, Dušica Vujaklija, Mile Šikić, Krešimir Križanović, Stjepan Gamulin

**Affiliations:** 1 Department of Pathophysiology, University of Zagreb School of Medicine, Zagreb, Croatia; 2 University of Zagreb School of Medicine, Zagreb, Croatia; 3 Research Department, University Hospital for Infectious Diseases “Dr. Fran Mihaljević”, Zagreb, Croatia; 4 School of Medicine, Catholic University of Croatia, Zagreb, Croatia; 5 Faculty of Medicine, University of Rijeka, Rijeka, Croatia; 6 St. Catherine Specialty Hospital, Zagreb, Croatia; 7 Forensic Science Program, Department of Biochemistry & Molecular Biology, The Pennsylvania State University, State College, PA, USA; 8 The Henry C. Lee College of Criminal Justice and Forensic Sciences, University of New Haven, West Haven, CT, USA; 9 Department of Pediatrics, University of Split Medical School, Split, Croatia; 10 Department of Pediatrics, Faculty of Medicine, Josip Juraj Strossmayer University of Osijek, Osijek, Croatia; 11 Medical School REGIOMED, Coburg, Germany; 12 Faculty of Dental Medicine and Health, Josip Juraj Strossmayer University of Osijek, Osijek, Croatia; dragan.primorac@svkatarina.hr * *; 13 Department of Biochemical Engineering, University of Zagreb Faculty of Food Technology and Biotechnology, Zagreb, Croatia; 14 Department for Nephrology, Internal Clinic, University Hospital Merkur, Zagreb, Croatia; 15 Department for Pathophysiology, Faculty of Medicine, Josip Juraj Strossmayer University of Osijek, Osijek, Croatia; 16 Bioinformatics group, Division of Molecular Biology, Department of Biology, Faculty of Science, University of Zagreb, Zagreb, Croatia; 17 Department of Innovative Diagnostics, Children's Hospital Srebrnjak, Zagreb, Croatia; 18 Department of Pediatrics, University Hospital Center Zagreb, Zagreb, Croatia; 19 Department of Pediatrics, University of Zagreb School of Medicine, Zagreb, Croatia; 20 Croatian Academy of Sciences and Arts, Zagreb, Croatia; 21 Department for Functional Genomics, Center for Translational and Clinical Research, University of Zagreb School of Medicine, University Hospital Centre Zagreb, Zagreb, Croatia; 22 Department of Neurology, University Hospital Centre Zagreb, Zagreb, Croatia; 23 Department of Microbiology, Croatian Institute of Public Health, Zagreb, Croatia; 24 Department of Microbiology, University of Zagreb School of Medicine, Zagreb, Croatia; 25 University of Zagreb School of Medicine and University Hospital Centre Zagreb (ret.), Zagreb, Croatia; 26 Department of Criminal Law, Faculty of Law, University of Zagreb, Zagreb, Croatia; 27 Department of Public Health, Faculty of Health Studies and Department of Social Sciences and Medical Humanities, Faculty of Medicine, University of Rijeka, Rijeka, Croatia; 28 Faculty of Humanities and Social Sciences, University of Zagreb, Zagreb, Croatia; 29 Greyledge Europe Ltd., Zagreb, Croatia; 30 Center for Applied Bioanthropology, Institute for Anthropological Research, Zagreb, Croatia; 31 International Burch University, Sarajevo, Bosnia and Herzegovina; 32 International Center for Applied Biological Sciences Ltd (ICABS), Zagreb, Croatia; 33 University of Split, University Department for Forensic Sciences, Split, Croatia; 34 University of Mostar, Law School, Mostar, Bosnia and Herzegovina; 35 Division of Physical Chemistry, Institute Ruđer Bošković, Zagreb, Croatia; 36 Faculty of Electrical Engineering and Computing, University of Zagreb, Zagreb, Croatia; 37 A*STAR Genome Institute of Singapore and National University of Singapore, Singapore

## Expansion of applied genomics in various fields urges the setting of a legal framework and ethical guidelines

Scientific discoveries and technological advancements have brought genomics into various spheres of modern society. Applied genomics significantly affected health care, biosafety, forensics, biotechnology, veterinary medicine, biodiversity, agriculture, information sciences, legislature, and other areas. However, applied genomics is characterized by the complexity of underlying technology, the abundance of information on genomic readouts, educational challenges, and ethical concerns stemming from the insights into the most intimate characteristics that biologically define an individual. Due to all this, there is an urgent need for immediate actions by concerned stakeholders to create rules and regulations for implementation of genomics in various fields of its applications and pave the way for its future development.

The Applied Genomic Committee of the Croatian Academy of Sciences and Arts formed working groups of leading Croatian experts to create a strategy that would facilitate the implementation of genomics in Croatia in the fields of biomedicine, biosafety and biosecurity, forensic medicine, biotechnology and bioinformatics, and computational biology with a special focus on ethical issues. As a result, the Croatian Academy of Sciences and Arts created and adopted a document entitled Applied Genomic Development Strategy for Croatia (1).

## Biomedicine

Identification of pathological gene variants (2-5), alterations in gene expression in human diseases (6-8), and studies of DNA structure (9,10) have been in the focus of researchers for a long time. Next-generation sequencing has opened new possibilities for identifying pathogenic gene variants (11). The section “Biomedicine” in the Strategy focuses on new fields of medicine that have arisen from applied genomics, such as pharmacogenomics and personalized/precision medicine (12-14). Next-generation sequencing, as the cornerstone of applied genomics, is becoming an important tool in the research and diagnostics of various diseases, such as cancer (15,16), rare diseases (17), and infectious diseases (18). The “Biomedicine” section also deals with the education of experts in genomic medicine (19,20), the integration between clinical practice and research, and the necessity of establishing a national center for genomics and legislature. Better collaboration between clinical and research institutions in genomics would result in multiple benefits in both areas, including faster implementation of personalized medicine, chance for professional growth, increased motivation and performance of experts, improved scientific outputs, and better technology transfer. The national center would foster the training of experts in the field of applied genomics in biomedicine. It would provide consultation services and facilitate technology transfer to smaller laboratories across Croatia. It is vital that institutions and experts in applied genomics in Croatia become part of European projects like 1+ Million Genomes, or infrastructure networks. One of such networks is the Elixir (https://elixir-europe.org/platforms), which is an important platform for sharing bioinformatics tools, training programs, and establishing contact with international experts. The Strategy encourages an interdisciplinary approach to applied genomics in biomedicine, especially in diagnostics and inclusion of different biomedical professions, such as medical doctors with relevant specializations, experts in biomedical technology, bioinformatics scientists, biomedical mathematicians, and technical personnel. There is a need to follow good practices and a legal framework produced by the European Union, which defines the specific roles in such teams.

## Biosafety and Bioprotection

Topics related to dangers associated with pandemics, bioterrorism, and natural disasters are addressed in the section “Biosafety and Bioprotection.” Valuable lessons learned from the COVID-19 pandemic were taken as a starting point for the preparation of mitigation measures for a potential similar future pandemic (21,22). Critical roles were played by Dr Fran Mihaljević University Hospital for Infectious Diseases in Zagreb, as the reference center for infectious diseases of the Republic of Croatia, and Dubrava University Hospital, as the COVID-19 hospital. The strategy promotes a multidisciplinary scientific, health care, and academic approach in dealing with applied genomics in biosafety and bioprotection. Croatia has an important role in defense against intentional or unintentional biological threats in warfare on the European continent due to its European Union and NATO membership and an important geopolitical and strategic position. The critical aspect of protecting its citizens from chemical, biological, radioactive, and nuclear weapons lies in the readiness to plan and act before, during, and after such incidents, including plans for evacuation and mass casualties. It is urgent to constitute Rapidly Deployable Outbreak Investigation Teams (RDOIT) and a team for intensive treatment of critical patients together with relevant hospital and laboratory infrastructure. The basic guidelines for creating the Biosafety and Bioprotection Strategy in the Republic of Croatia were made according to the Homeland Security System Act. Objectives include creating a network of institutions for the defense against bioterrorism and natural biological disasters, advancing their infrastructure capacities, training of personnel, defining funding and creating sustainability, promoting cooperation with relevant international institutions, and creating plans and responsibilities in the event of quarantine diseases.

## Forensic Genomics

Forensic genomics is a rapidly growing field that employs multiple approaches, including sequencing mitochondrial and nuclear DNA to identify individuals (23-27). The Strategy contains a section called “Forensic Genomics” that focuses on important issues in applied genomics in Croatia, including the identification of victims of Homeland War in Croatia, implementation of new technologies, cooperation among relevant institutions, international collaboration, education of experts and building teams, data exchange, ethical principles, and development of legal framework. This section focuses explicitly on the latest advancements of many new technologies and elaborates on the necessity to stimulate collaboration among institutions concerned with forensics.

To retain one of the leading positions in forensic genomics, Croatia must actively develop and apply additional new technologies in the field, which should be systematically integrated and joined with already routinely used procedures, such as next-generation sequencing, forensic phenotyping, forensic plant and animal DNA analyses, artificial intelligence, molecular autopsy, etc.

The development of all these new methods will also significantly help continue the most important mission of Croatian society – identifying Homeland War victims and applying forensic genomics according to the highest ethical standards.

The interdisciplinary approach and interaction between scientific/academic institutions and official forensic governmental institutions are paramount for the successful application of forensic genomics. Additionally, cooperation with leading international forensic institutions is necessary since international crime, human trafficking, bioterrorism, etc, cannot be effectively combated without data exchange. Future education in this field should be focused on the most advanced training of crime scene and laboratory experts. Only in this way, the Croatian Forensic Genomics Society will be able to provide all required information to investigative and judicial bodies, as well as to maintain the highest international ranking that Croatian scientists have reached in this scientific field.

## Ethical principles of applied human genomics

Genetic testing data may result in various forms of discrimination against the affected individual and his or her family. This is especially true for wide genomic analyses that may discover secondary findings or accidental gene variants that increase the likelihood of a disease occurring in the descendants of an individual who may have a mild form of the disease or not have the disease at all (28,29). The working group on ethical concerns in applied genomics identified several key issues that need to be considered in applied genomics, especially following whole-genome analyses, which agrees with previously published documents (30-33). The most important current ethical principles that need to be followed in applied genomics are based on the general principles of humanity, freedom, and autonomy of the individual in decision-making, equality, solidarity, social justice, the right to privacy, responsibility, security, knowledge, the right to information, and general respect for human rights. These principles lie beyond scientific and technological capabilities and reach. Applied genomics procedures ought to be accessible to everyone and may be carried out only for medical or scientific purposes. Human genome modification procedures may only be conducted for preventive and therapeutic purposes so that the altered genes are not passed on to the offspring. Cloning or creating genetically identical humans is considered ethically unacceptable and neither is creating beings from different types of living creatures that include parts of human genes. Only trained and certified experts in accredited laboratories and institutions may conduct procedures in the field of genomics. Anyone who suspects a violation of ethical principles in applied genomics should report the information to the competent authorities. Therefore, continuous wide public debate on the matter is of common interest and should be firmly encouraged.

## Biotechnology

Modern biotechnology is one of the leading technologies of the 21st century, providing bio-based commodities, services, and, most importantly – knowledge, with two essential consequences for humankind: preserving biodiversity and improving the quality of life (34-37). Innovative and integrated biotechnologies have inevitably readjusted into biorefineries (38) and beyond, diffused in bioeconomy and circular economy, and thus tackled key challenges of contemporary society – climate change, health care, and geopolitical events. Therefore, the “Biotechnology” section of the Strategy distinguishes all stakeholders of modern society, which is of utmost importance especially in crises, as we have learned recently during the COVID-19 pandemic. Novel biotechnological products and services, especially in health care, are to be applied only when clearly and unambiguously defined by law and after consideration of ethical and moral issues. Thematic areas of modern biotechnology (agroecology, food production, and supply systems, the forest value-added chains, management of freshwater and marine coastal resources, bioenergetics, advanced biochemicals, pharmaceuticals and biomaterials, and education in the field of biotechnology) clearly depict the status of available resources and new directions of their multiple utilization with zero waste.

## Bioinformatics and Computational Biology

Although listed as the last, the “Bioinformatics and Computational Biology” section is the key segment and a bottleneck in applied genomics due to the critical shortage of such experts in Croatia. One of the important objectives of experts in bioinformatics and computational biology is to create important tools for various forms of big data analyses produced by different “omics” approaches (39,40). As in other countries, data sharing and safeguarding are crucial for maximizing the exploitation of genomic data in Croatia (41). Sequencing technology has altered the approach to scientific work in the natural sciences, biotechnology, and biomedicine, and caused changes in diagnostic and clinical medicine, veterinary medicine, and agronomy. New insights into genetic data from individuals and entire populations create possibilities for treating previously untreatable diseases, driving innovations and progress in society. Successful application and use of genomic data relies on digital technologies, namely, computer methods and data sciences focused on storage, analysis, and visualization technologies of large amounts of data (big data). The Strategy explains the importance of establishing a national computer infrastructure center that would allow a higher degree of synergy and collaboration among Croatian researchers. The center would promote the creation of protocols that enable structured and consistent collection and storage of genetic data and associated metadata, especially human genetic, clinical and personal data. It would foster the development of cutting-edge data science technologies, including artificial intelligence and machine learning, stimulate the establishment of support systems for professional users, and promote the creation of computer tools and procedures for end-users, as a help in the interpretation of the results of genomic tests. Establishing protocols for data protection, education of experts, management of computer infrastructure, and communication with the public would also be tasks of this center.

## Concluding remarks

The Applied Genomics Development Strategy for Croatia, an official document of the Croatian Academy of Sciences and Arts, delineates key aspects of applied genomics in Croatia and paves the way for its future development. These include the education of experts, development of infrastructure, rational use of resources, the establishment of a national institute of applied genomics, collaboration among relevant institutions, and integration of efforts with researchers. A growing number of human genome analyses are performed each day, and new possibilities of genomic manipulation increase the need of our society to develop a legal framework and ethical guidelines. These recommendations would allow unhindered development of applied genomics, but also prevent malicious alterations of genomic properties of living organisms and protect the privacy of an individual with a genetic predisposition to certain diseases. The Strategy identifies genomics as the cornerstone of the concept of personalized/precision medicine, which allows provision of a medical care tailored to meet the specific needs of an individual. Moreover, the Strategy also paves the way for the development of forensic genomics as an indispensable tool in fighting crime and identifying war victims, including those from Homeland War in Croatia. Given the persistent threat from potentially devastating pandemics and bioterrorism acts in the future, the Strategy provides a framework of actions and plans to protect human lives and the economy, which makes it a vital document for national security. Applied genomics opens new possibilities for the growth of our economy by improving biotechnological processes and bioinformatics tools, promoting the efficient use of energy, sustainable industry, and environmental protection. Like the similar strategies of development of applied genomics that have been adopted by technologically advanced countries, this Strategy identifies key elements that will promote the well-being of citizens and help us in the transformation into a modern society.
